# Supplementary material for: Physical activity and handgrip strength in patients with mild, moderate and severe haemophilia: Impacts on bone quality and lean mass
Source: PLoS One. 2025 Mar 26;20(3):e0319951. doi: 10.1371/journal.pone.0319951 (PMC11940556; doi:10.1371/journal.pone.0319951)
Supplement: S1 Fig — Minimal data set for boxplots showing the different severity phenotypes regarding physical activity, bone quality, lean mass and handgrip strength. (DOCX) [file pone.0319951.s001.docx]

| Variables | Severe  (n=132) | Moderate  (n=46) | Mild  (n=45) | Total  (n=223) |
| --- | --- | --- | --- | --- |
| Steps per day  M±SD Median [Q1, Q3] | 7509 ± 3851  7095 [4757, 9801] | 8653 ± 4249 7040 [5530, 11472] | 8446 ± 3764 8466 [5219, 10785] | 7934 ± 3935 7392 [4981, 10597] |
| Activity per day (minutes) M±SD Median [Q1, Q3] | 51 ± 58  38 [17, 68] | 52 ± 54 42 [12, 82] | 64 ± 65  42 [25, 85] | 54 ± 53 42 [17, 72] |
| Handgrip strength (kg) M±SD Median [Q1, Q3] | 38.6 ± 11.5 38.0 [30.6, 48.0] | 43.9 ± 7.0 43.3 [39.6, 50.4] | 43.6 ± 7.6 41.4 [38.5, 49.9] | 40.7 ± 10.2 41.0 [35.1, 48.6] |
| Bone mineral density (g/cm2) M±SD Median [Q1, Q3] | 0.795 ± 0.144 0.774 [0.696, 0.887] | 0.832 ± 0.132 0.818 [0.735, 0.932] | 0.915 ± 0.241 0.880 [0.743, 1.001] | 0.827 ± 0.172 0.807 [0.710, 0.923] |
| Trabecular bone score M±SD Median [Q1, Q3] | 1.408 ± 0.130 1.448 [1.330, 1.500] | 1.379 ± 0.124 1.397 [1.302, 1.480] | 1.404 ± 0.191 1.446 [1.337, 1.549] | 1.401 ± 0.142 1.431 [1.329, 1.499] |
| Lean mass (g)  M±SD Median [Q1, Q3] | 55269 ± 8792 53532 [49488, 59784] | 58637 ± 6775 59232 [52389, 61966] | 57488 ± 5938 58087 [53399, 61825] | 56411 ± 7976 55722 [50877, 61236] |

S1 Figure 1: Minimal data set for boxplots showing the different severity phenotypes regarding physical activity, bone quality, lean mass and handgrip strength
