# Supplementary material for: Physical activity and handgrip strength in patients with mild, moderate and severe haemophilia: Impacts on bone quality and lean mass
Source: PLoS One. 2025 Mar 26;20(3):e0319951. doi: 10.1371/journal.pone.0319951 (PMC11940556; doi:10.1371/journal.pone.0319951)
Supplement: Attachment — Submitted filename: Response to Reviewers pdf.pdf [file pone.0319951.s002.pdf]

Reviewer #1:

1. The authors report on 223 included patients, but less than half of them had a handgrip strength test and not all returned the diary. Of those who returned the diary, some reported that they did not perform any sports.
   A table that lists all the investigations with numbers of patients in whom the parameters were collected should be given. How did the authors handle missing values in the statistical evaluation?
   Thank you for this valuable input. It is necessary that the sample sizes are clearly determined. We now included the number of patients for each parameter in the legends of the figures (see figure 1 & 2). As this is a cohort study with multiple outcome parameters, it is challenging to completely avoid missing data. For the statistical analysis, we addressed this by excluding the missing data to ensure the robustness of our findings.

2. Provide numbers of hemophilia A and B in table 1.
   Thank you very much for this advice. We included the respective numbers in table 1.

3. The joint score HJHS differed – as expected – between hemophilia severities but was not fully included in the analyses. Was there any associations of outcomes (BMD and TBS) with HJHS?
   Thank you for this question. The data of the present manuscript were part of a large investigation. We previously published data regarding the HJHS and BMD, indicating that HJHS predicts BMD (B= −0.002; 95% CI: −0.004, −0.001; p = 0.02). To avoid duplication of data presentation, these results were not mentioned in the present manuscript again.

4. Table 2: It would be fine to also include percentage values.
   Thank you for your valuable feedback. The percentage values are now included in table 2.

5. Page 8. Line 4: …post-hoc testing of joint score. Why post-hoc testing? Please explain.
   Thank you for your comment. To be more precise, we added "Bonferroni" post-hoc-testing in the text (see page 8, line 160) As the Kruskal-Wallis-Test showed a significant difference of p=0.001 for the severity phenotype regarding joint score, a post-hoc testing was used for (1) the calculation pairwise comparisons and (2) to adjust the p-value to minimize the type-I error rate.

6. Page 17, first line: "…still being gentle the joints" Please clarify or correct.
   Thank you for your valuable feedback. To be clearer, we adjusted the sentence to: "while minimizing excessive mechanical stress on the joints" (see page 18, line 419).

Reviewer #2:

1. Since only the HJHS significantly differed between the different degrees of severity, it would be interesting to know if this had an influence.
   Thank you very much for this comment. As stated on page 13, line 322 as well as page 14, line 330, the HJHS was significantly correlated with objective PA and handgrip strength. Indicating that the worse the joint status, the lower the handgrip strength and the lower the objective PA. For subjective PA, no significant correlations were found. Furthermore, the data of the present manuscript were part of a large investigation. We previously published data regarding the HJHS and BMD, indicating that HJHS predicts BMD (B= −0.002; 95% CI: −0.004, −0.001; p = .02). To avoid duplication of data presentation, these results were not mentioned in the present manuscript again (see doi: 10.1016/j.rpth.2024.102624 & doi: 10.1111/hae.15091).

2. As there is no healthy control group, what would the parameters look like in a population without haemophilia?
   Thank you for your question. Depending on the outcome parameter, we included comparisons to the non-haemophilic European population (PA) (see page 14, line 334, and 337). Regarding bone quality and body composition parameters, the comparisons to a healthy population were already stated in previously published papers, so that we did not mention these data again to avoid duplication (see doi: 10.1016/j.rpth.2024.102624 & doi: 10.1111/hae.15091).

3. Not all patients followed the sports activities. What were the reasons for this?
   Thank you for highlighting this point. Some haemophilia-specific reasons might be presence of pain, restriction of joint function or fear of exercise-induced bleeding events (as stated on page 14, line 338-341). However, an evaluation of possible barriers to perform sports was not conducted within this research. Of course, there might also be non-haemophilia specific barriers to perform sports. We added this paragraph in the discussion section including a respective reference (doi: 10.3390/ijerph18115810; see page 14, line 341-343).

4. Could some information on social status and occupation be added?
   This is a very good idea, thank you. Unfortunately, within this research project we did not gather data on social status/occupation of the patient. We included this aspect in the limitations section (see page 17, line 405-407).

5. There is a wide age range. Is the younger population different from the older one?
   Thank you for this valuable question. Based on an additional correlation analysis we could demonstrate that age does not impact handgrip strength nor subjective PA. Objective PA was weakly correlated with age (rho= -0.164, p=0.014). We added this finding in the results section (page 13, line 303-306) and discussed this in on page 14, line 344-347 including a respective reference (doi: 0.1186/s12877-021-02188-9).
